# Organizational factors associated with community therapists’ self-efficacy in EBP delivery: The interplay between sustainment leadership, sustainment climate, and psychological safety

**DOI:** 10.1177/26334895221110263

**Published:** 2022-07-04

**Authors:** Y. Vivian Byeon, Anna S. Lau, Teresa Lind, Alison B. Hamilton, Lauren Brookman-Frazee

**Affiliations:** 1 Department of Psychology, 8783 University of California, Los Angeles, Los Angeles, CA, USA; 2 Department of Child & Family Development, San Diego State University, San Diego, CA, USA; 3 229241 Child and Adolescent Services Research Center, San Diego, CA, USA; 4 Department of Psychiatry and Biobehavioral Sciences, 8783 University of California, Los Angeles, Los Angeles, CA, USA; 5 Center for the Study of Healthcare Innovation, Implementation, & Policy, VA Greater Los Angeles Healthcare System, Los Angeles, CA, USA; 6 Department of Psychiatry, University of California, San Diego, La Jolla, CA, USA

**Keywords:** inner context, self-efficacy, sustainment leadership, sustainment climate, mental health workforce, organizational climate, psychological safety

## Abstract

**Background:**

Inner context organizational factors proximally shape therapist experiences with evidence-based practice (EBP) implementation and may influence therapist self-efficacy, which has been linked to sustained use of EBPs in community mental health settings. Research has primarily focused on constructs such as implementation leadership and climate. However, the effects of such factors may depend upon other inner context dimensions, such as psychological safety. Psychologically safe environments are conducive to taking risks, speaking up about problems, and requesting feedback and may promote therapist self-efficacy during implementation. This study examines whether organizational sustainment leadership and sustainment climate relate to therapist EBP self-efficacy only under conditions of psychological safety.

**Methods:**

Data were collected from 410 clinicians in 85 programs during the sustainment phase of a system-driven implementation of multiple EBPs in children's mental health services. Therapists reported on their organization's sustainment leadership, sustainment climate, psychological safety, and their own self-efficacy in delivering specific EBPs. Multilevel regression analyses were conducted to account for nested data structure.

**Results:**

Among program-level variables, sustainment leadership and psychological safety both significantly predicted therapist self-efficacy. However, there were no significant interactions between program-level sustainment climate and psychological safety. Exploratory post-hoc analyses revealed a significant interaction between program-level sustainment leadership and therapist-level perceptions of psychological safety such that that the conditional effect of psychological safety on EBP self-efficacy was significant at high levels of sustainment leadership, but not at low or average levels.

**Conclusion:**

We noted independent links between sustainment leadership, organizational psychological safety and therapists feelings of confidence and mastery with EBPs. Therapists’ individual perceptions of psychological safety were linked to self-efficacy only in programs with high sustainment leadership. Thus, sustainment leadership and psychological safety may both represent implementation intervention targets, but it may not be critical to assess for perceptions of psychological safety before deploying organizational leadership strategies.

**Plain language abstract** Therapist self-efficacy is a therapist's belief that they are capable, knowledgeable, and skilled enough to deliver evidence-based practices (EBPs), and is thought to promote improved clinical and implementation outcomes, such as therapists’ sustained use of EBPs. Conditions within community mental health organizations may influence therapists’ sense of EBP self-efficacy. Leaders’ support and expectations for EBP implementation, and collective staff perceptions about the organization's climate to support EBPs are linked to positive therapist attitudes and EBP adoption. However, less is known about how these implementation-specific organizational factors associated with therapist EBP self-efficacy in the long-term, and how this may depend on general workplace conditions. Specifically, psychologically safe environments – where therapists feel safe taking risks such as asking questions, admitting mistakes, and trying new skills – may be needed to promote self-efficacy when therapists are tasked with learning and using complex multi-component EBP innovations. The current study tested the prediction that leader-driven and program-wide focus on EBP sustainment may promote therapist EBP self-efficacy only in organizations where conditions for learning are psychologically safe. Our findings confirmed that fostering strong sustainment focused leadership and psychologically safe environments may each be important for increasing therapists’ EBP self-efficacy. The model results suggested that individual therapist perceptions of psychological safety were more strongly related to EBP self-efficacy in programs with greater implementation leadership. Findings suggest the importance of increasing EBP leadership behavior to fully potentiate other facilitating conditions for therapist learning in the sustainment phase of EBP implementation initiatives.

Over the past 50 years, numerous evidence-based practices (EBPs) have been developed and identified as effective treatments for youth with psychiatric disorders (Williams & Beidas, 2019). However, access to EBPs is highly variable in community mental health settings (Bruns et al., 2015; Wang et al., 2010). In response, public behavioral health systems have pursued scale-up efforts to promote the implementation of multiple EBPs. These systems provide training and consultation for community therapists, as well as incentives or requirements such as collection of outcome measures, enhanced reimbursement for EBP use, and public recognition of EBP providers to support EBP learning, adoption and use (Regan et al., 2017; Stewart et al., 2018). As these efforts have increased, so has the pressure and expectation for community mental health therapists to learn these EBPs and for organizations to support and provide resources for them. Guided by the Exploration, Preparation, Implementaiton, and Sustainment (EPIS) framework, (Aarons et al., 2011) in the past decade, implementation research has examined inner context elements, such as therapist and organizational factors that facilitate the adoption and implementation of EBPs, in different outer context settings in behavioral health systems across the United States (Beidas et al., 2015, 2019; Hoagwood et al., 2014; Lau & Brookman-Frazee, 2016; Nakamura et al., 2014). This research has mostly focused on use of EBPs with fidelity after the implementation phase as an indicator of successful implementation. However, as many systems have already significantly invested in EBP training and implementation strategies, it becomes important to explore inner context factors and structures that predict sustained EBP use. A readily assessed therapist factor that has been shown to relate to sustained delivery of EBPs (Lau et al., 2020) is therapist self-efficacy with EBPs.

 Self-efficacy – the belief that one is capable, knowledgeable, and skilled enough to complete a task (de Vries et al., 1988) – has robust links to future behavior change in many workplace sectors, such as health care (Holden, 1991), social work (Collins-Camargo & Royse, 2010; Montcalm, 1999), and technology (Hsu et al., 2009). In mental health service settings, therapist EBP self-efficacy is hypothesized to be an important factor in EBP delivery across many phases of implementation, including preparation, implementation, and sustainment (Aarons et al., 2012; McLeod et al., 2018). Among community therapists within a system-driven implementation effort of multiple EBPs, therapist-reported self-efficacy with a given EBP was associated with decreased likelihood of discontinuation of that EBP (Lau et al., 2020) and increased observed extensiveness of EBP strategy delivery in session (Lind et al., 2021), indicating that self-efficacy may be an important target for EBP sustainment efforts. Self-efficacy is also hypothesized to be an important therapist factor in improving clinical outcomes. In a randomized controlled trial of cognitive processing therapy, therapist self-efficacy ratings were related to patient clinical improvement. In addition, among therapists with low baseline self-efficacy, receipt of consultation resulted in better client outcomes (Pace et al., 2020). These findings suggest that organizations may be able to improve care and EBP implementation outcomes by investing in strategies that promote therapist self-efficacy.

To date, research on inner context organizational factors related to EBP implementation has largely focused on improving the EBP implementation specific components, such as EBP implementation leadership and climate (Aarons et al., 2009; Williams et al., 2020). Strong implementation leadership is characterized by leaders who are knowledgeable about, supportive of, and perseverant through challenges associated with EBP implementation (Aarons et al., 2014). Positive EBP implementation climate is denoted by employees’ shared perception of the importance of EBP implementation within the organization (Ehrhart, Aarons, et al., 2014). Both implementation climate and leadership have been posited to improve EBP implementation through supporting therapist acquisition of EBP knowledge and skills through educational implementation strategies, removing barriers, and enhancing motivation to EBP use (Aarons & Sommerfeld, 2012; Jacobs et al., 2014). Previous studies have also found that higher levels of EBP implementation climate and leadership are associated with more positive therapist EBP attitudes, increased EBP knowledge, and increased use of EBP techniques (Powell et al., 2017; Williams et al., 2020). These findings suggest that organizational leaders’ persistent and proactive promotion of EBPs can improve provider implementation outcomes in community settings. It should also be noted that implementation climate is theorized to mediate the relationship between implementation leadership and employee behavior (Ehrhart, Schneider, et al., 2014). However, less is known about how these factors interact to influence EBP self-efficacy. Organizational commitment to training and supportive leadership have been posited to influence individual self-efficacy regarding EBPs, (Cannon-Bowers et al., 1995; McLeod et al., 2018) but links to this outcome have yet not been empirically tested. Furthermore, little data is available concerning how these organizational factors operate in the sustainment phase of implemenation.

There is also a need to examine inner context organizational characteristics (beyond those specific to EBP implementation) that may also determine the outcomes of implementation leadership and climate. For example, one study by found a significant interaction between implementation climate and molar climate, such that organizations with positive molar climates and high levels of implementation climate predicted the highest EBP use for their clinicians (Williams et al., 2018). Given considerable organizational variation in workplace climate, it may be important to identify inner context organizational characteristics that have implications for the impact of implementation strategies and interventions. One organizational factor that may be important to consider is psychological safety, i.e., the degree to which employees perceive that their workplace is conducive to taking interpersonal risks, such as speaking up or asking for help (Edmondson, 1999). Psychologically safe environments, where people feel accepted, supported, and trusted, promote sharing of information (Collins & Smith, 2006), experimenting with new skills (Baer & Frese, 2003), and upward communication to management (Edmondson & Lei, 2014) - all processes that underlie workplace effectiveness and organizational learning. While psychological safety can be studied at multiple levels (e.g., individual-, group-, organizational-), it is most commonly studied at the group- and organizational-levels (Edmondson & Lei, 2014) Group- and organizational-level psychological safety has been found to promote employee learning and behavior change in corporate settings and complex work environments such as health care and education sectors (Edmondson, 2003; Edmondson et al., 2016; Nembhard & Edmondson, 2006; Singer et al., 2009). Yet, organizational-level psychological safety has yet to be examined in relation to EBP implementation or sustainment outcomes in behavioral health settings (Williams & Glisson, 2020).

Organizational interventions aimed at improving implementation leadership and climate have also implicated organizational-level psychological safety as an essential mechanism to ready organizational contexts for successful EBP implementation (Aarons et al., 2017; Glisson et al., 2010, 2016). Fostering psychologically safe environments may be especially important for frontline therapists who are responsible for delivering multiple EBPs in resource-limited community mental health settings serving high-need populations. As part of EBP implementation initiatives, therapists have been asked to perform new behaviors (e.g., learning and using new techniques, asking for and receiving supervision and performance feedback), which can be viewed as risky because they could open up individuals to negative evaluation. Even if organizations provide clear messages about the priority of EBPs through its leadership and climate, therapists may hestitate to take risks that promote learning if they perceive that the organization is not psychologically safe. In contexts where client well-being is at stake and complex multicomponent behavioral interventions are being introduced, it is critical for therapists to feel safe to grow from experience with the innovation, learn from mistakes, and benefit from fidelity monitoring (Edmondson, 2003; Edmondson et al., 2016). Psychologically safe environments are conducive to improving staff learning and performance by setting the conditions to try out practice innovations without negative consequences (Edmondson & Lei, 2014). EBP self-efficacy is theorized to change as therapists gain experience applying skills and knowledge and benefitting from training and consultation (McLeod et al., 2018). Environments in which therapists are willing to pose questions, discuss errors, and learn from their mistakes set the stage for therapists to be comfortable engaging in active learning components of training and activities such as fidelity monitoring and performance feedback (Dorsey et al., 2013; Lucid et al., 2018) ultimately impacting their sense of self-efficacy. Additionally, a psychologically safe work environment may also encourage therapists to share information and problem-solve challenges in implementation at the local level, further boosting agency and self-efficacy.

Thus, a critical unexamined question is whether the effects of implementation leadership and climate on EBP self-efficacy may depend on psychological safety. In particular, a concerted emphasis on EBP implemenation at the organizational-level may have differential effects, depending upon whether therapists perceive the environment as safe for learning practice innovations. Therefore, the current study examines the interplay of sustainment leadership, sustainment climate, and organizational-level psychological safety in predicting therapists’ self-efficacy with EBP delivery.

Within the context of a system-driven implementation of multiple EBPs for children in the Los Angeles County Department of Mental Health (LACDMH; Regan et al., 2017), the current study examined how organizational sustainment leadership and EBP sustainment climate are associated with therapist perceptions of EBP self-efficacy and whether these relationships may be moderated by organizational-level psychological safety (See Figure 1). Moving forward, this is described as “program-level” psychological safety in the present study to accurately describe the organizational structure of this context in which therapists are aligned to programs within specific agencies. Specifically, we hypothesized that more positive sustainment leadership and higher levels of sustainment climate would predict higher therapist self-efficacy for EBPs under conditions of program-level psychological safety for learning. Under positive conditions for learning, an organizational climate focused on the importance of sustaining EBPs and sustainment leadership may provide the conditions under which therapists can develop self-efficacy. However, a high-stakes focus on EBPs through implementation-specific workflows and leadership messaging may inhibit therapist EBP self-efficacy when they perceive the organizational climate is not safe for learning.

**Figure 1.**
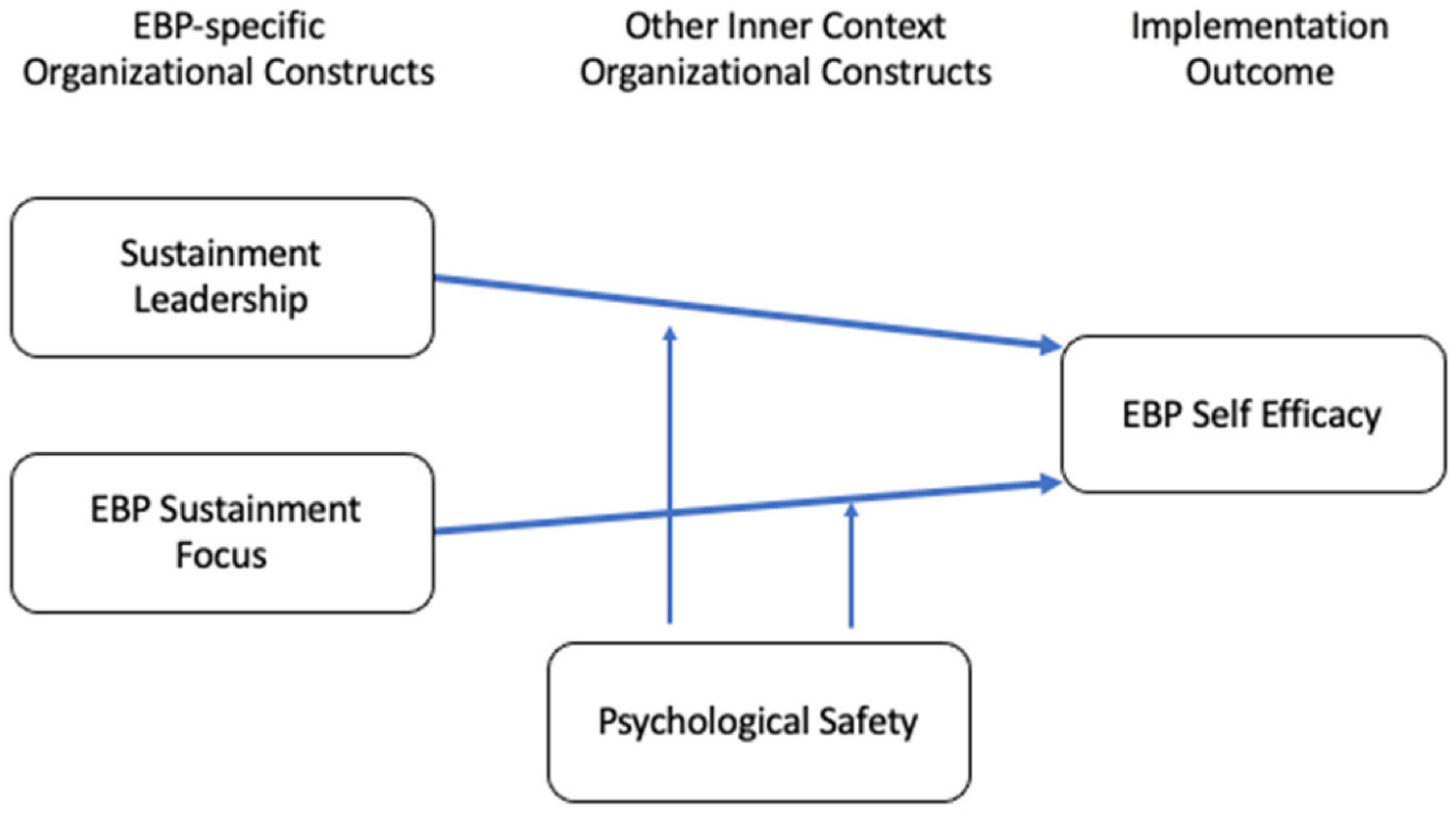
Conceptual model of the effect of inner context organizational constructs on EBP self-efficacy.

## Methods

### Setting

Data for the current study were collected via an online survey fielded in 2019 as part of the Knowledge Exchange on Evidence-Based Practice Sustainment (4KEEPS) study (Lau & Brookman-Frazee, 2016). The 4KEEPS study is an observational study that examined therapist and organizational factors associated with sustainment of multiple EBPs in the context of a system-driven implementation in Los Angeles County. LACDMH is the largest county mental health department in the United States, serving more than 250,000 residents annually (Los Angeles County Department of Mental Health, n.d.). In 2010, the LACDMH launched the Prevention and Early Intervention (PEI) program which facilitated EBP implementation by furnishing EBP training and consultation and offering agencies reimbursement for the delivery of EBPs. The 4KEEPS survey administered in 2019 focused on nine EBPs that were among the most commonly delivered within PEI: Incredible Years Parenting Program (IY), Interpersonal Therapy (IPT), Parent Child Interaction Therapy (PCIT), Child-Parent Psychotherapy (CPP), Managing and Adapting Practice (MAP), Seeking Safety, Triple P Positive Parenting Program (Triple P), Trauma Focused Cognitive Behavior Therapy (TF-CBT), and Cognitive-Behavioral Intervention for Trauma in Schools (CBITS).

### Participants

The participants for the current study included 410 therapist respondents from 85 programs that were contracted by LACDMH to deliver EBPs through PEI in children's mental health services. Eligible participants included any therapist who billed for psychotherapy services to at least one of the nine commonly delivered EBPs to youth. Most therapists were female (91.22%), had a master's degree (91.71%), were not licensed (66.10%), and had CBT or behavioral theoretical orientation (63.90%). The mean age and SD were 34.47 and 7.67, respectively. This sample was racially and ethnically diverse, with 53.66% identifying as Hispanic, 20.00% as white, and 26.34% as other ethnic minorities (e.g., Asian, Black, etc.). Table 1 presents the therapist demographics of the sample.

**Table 1.** Descriptive statistics (N = 410).

Variable	Mean (SD)	Percent
Gender (Female)		91.22%
Race/Ethnicity		
Non-Hispanic White		20.00%
Hispanic		53.66%
Other Ethnic Minorities		26.34%
Education		
Less than Master's degree		1.95%
Master's Degree		91.71%
Doctoral Degree		6.34%
Licensure Status		
Licensed		33.90%
Not Licensed		66.10%
Theoretical Orientation		
CBT or behavioral		63.90%
Humanistic		4.88%
Family Systems		12.93%
Psychodynamic		6.58%
Eclectic		9.51%
Other		2.20%
Age	34.47 (7.67)	
Hours per week of direct service	23.01 (7.09)	

### Procedure

Data collection for this survey occurred in May through August 2019. Participants were recruited by gathering therapist and program leader contact information from leaders of organizations that had participated in a prior therapist survey from the 4KEEPS project fielded in 2016 (Barnett et al., 2017). Of 82 agencies eligible for youth PEI service contracts, 37 (45.1%) provided contact information for therapists employed in 87 distinct programs. All program leaders and therapists were then invited by email to participate in the online survey. Email invitations were sent directly to 1447 individuals. The survey was completed by 448 therapists, 113 program leaders, and 33 individuals who identified as both therapist and program leader, leading to a response rate of 41.1%. This is within the range of previous surveys of previous community mental health therapists (Hawley et al., 2009). We are unable to produce specific response rates for program leaders and therapists because we do not know the which of the 1447 individuals were therapists or leaders. Participants received a $40 electronic gift card for completing the survey. All procedures for this study were reviewed and approved by the Institutional Review Boards at LACDMH, University of California, Los Angeles, and University of California, San Diego. The study was granted a waiver of signed consent and a written information sheet was provided to each participant on the first page of the online survey. For the current study, 38 therapists were excluded from the analyses because they did not provide complete data for the dependent variable, resulting in N = 410 as described above. Program leader data were not included in the present study due to the focus on front-line therapist perceptions of organizational constructs, which is the convention in the literature (Weiner et al., 2011).

### Measures

#### Psychological safety scale

Psychological safety was measured using an adapted 4-item version of the Psychological Safety Scale (Edmondson, 1999). Therapists reported to what extent each of the four statements applied to their organizations on a 5-point Likert scale from “Strongly disagree” (1) to “Strongly agree” (5). Example statements include “It is safe to take a risk in this program,” and “Therapists who work here are able to bring up problems and tough issues.” “Program” referred to the therapist's organization and work setting. The adapted scale differs from the original scale in two ways: 1) text in the items were modified from “team” and “someone” to reflect the current context (e.g., “program” and “therapists”), 2) we removed 3 items from the original scale to focus on items that were relevant to the EBP implementation context. The content of the 4 included items assessed climate that promoted safety in learning a practice innovation (e.g., “It is difficult to ask other staff in this program for help” and “Therapists who work here are able to bring up problems and tough issues) rather than a more general atmosphere of interpersonal trust and acceptance (e.g., “People on this team sometimes reject others for being different” and “Working with members of this team, my unique skills and talents are valued and utilized”). In the present study, internal consistency for the scale was acceptable (α = .72) and there was significant program-level variance (ICC = .18).

#### Sustainment leadership scale

Sustainment Leadership was measured using an adapted 9-item version of the psychometrically validated Implementation Leadership Scale (ILS) (Aarons et al., 2014). This scale assesses the degree to which leaders are knowledgeable (“Leaders in my program know what they are talking about when it comes to EBPs”), supportive (“Leaders in my program support staff efforts to learn more about EBPs”) and perseverant (“Leaders in my program persevere through the ups and downs of sustainment of EBPs”) in implementing EBPs. The adapted version differs from the original ILS in three ways: 1) the proactive subscale was removed due to concerns about overburdening participants and lack of relevance for the current study's system-driven sustainment context, 2) “leaders in my program” was included in each item rather than the name of each supervisor, and 3) the items were reworded to reflect EBP sustainment rather than implementation (“Leaders in my program recognizes and appreciates employee efforts toward successful sustainment of EBPs”). Therapists reported to what extent each statement applied to their program's leadership on a 5-point Likert scale from “Not at all” (1) to “To a very great extent” (5). The scale demonstrated excellent internal consistency (α = .97) and significant program-level variance (ICC = .20).

#### EBP sustainment climate

Perceptions of organizational climate on EBPs were measured using a 3-item subscale of the Sustainment Climate Scale, which was adapted from the Implementation Climate Scale (ICS) (Ehrhart, Aarons, et al., 2014). The subscale assesses the degree to which organization members prioritize EBP use and sustainment (“One of this program's main goals is to use evidence-based practices effectively”) and only one word was adapted from the subscale item of the ICS to capture sustainment rather than implementation (“People in this program think that the sustainment of evidence-based practices is important”). Therapists reported to what extent each statement applied to their program on a 5-point Likert scale from “Not at all” (1) to “To a very great extent” (5). Internal consistencies were excellent (α = .93), and the intraclass correlations indicating program-level variance was substantial (ICC = .15).

#### Therapist report of EBP self-efficacy

Therapist self-efficacy in delivering EBPs was measured using 4 items that included two from a previously published EBP-specific self-efficacy measure (Lau et al., 2020). The prior two-time measure assessing confidence in delivering each of the EBPs the therapist is currently using with clients (“I am confident in my ability to implement this EBP,” “I am well prepared to deliver this EBP even with challenging clients”). Following a qualitative analysis of in-depth interviews with community therapists, two items were added to reflect additional concepts related to self-efficacy – feeling knowledgeable about (“I am knowledgeadble about this EBP”) and understanding the EBP model (“I have a good understanding of all of the components of this EBP”). Therapists rated themselves on a 5-point Likert scale from “Not at all” (1) to a “Very great extent” (5). Therapists completed this scale for each EBP they were currently using with clients at their agency. On average, therapists reported using 1.95 EBPs with clients (SD = 1.10). The four items were averaged to create a composite score. Internal consistency for this scale was excellent (α = .94). Also, we calculated ICCs at level 2 (.32) and level 3 (.08) for self-efficacy, indicating that between-therapist, and between-program differences do account for variability in reported self-efficacy.

### Statistical analyses

This study intended to examine 1) if sustainment leadership and EBP sustainment climate were associated with therapists’ self-efficacy for EBPs and 2) whether psychological safety moderated the relationships in the first aim. Multilevel models were conducted to account for the nested data structure, with EBPs (level 1; n = 932) nested within therapists (level 2; n = 410) nested within programs (level 3; n = 85). For program-level variables, therapist reported perceptions were aggregated to the program-level by taking the mean score of all therapists in a particular program.To justify aggregation, we calculated interrater agreement (i.e., r_wg(j),_) for each program-level variable (LeBreton & Senter, 2008). The mean r_wg(j)_s for program-level psychological safety (r_wg(j)_ = .73), sustainment leadership (r_wg(j)_ = .74), and sustainment climate (r_wg(j)_ = .76) all demonstrated strong agreement (.71 < r_wg(j)_ <.91) according to LeBreton & Senter's revised standards for interpreting interrater agreement estimates (2008). In terms of the models, first, we ran a multilevel regression with random intercepts using the “mixed” command and maximum likelihood estimator in Stata with two level 3 interactions with program level psychological safety (i.e., psychological safety x sustainment leadership, psychological safety x EBP sustainment climate) as predictors and EBP self-efficacy (at level 1) as the outcome. Based on previous research on therapist-level factors associated with similar therapist-reported EBP implementation outcomes (Lau et al., 2020), we included the following therapist-level covariates in our analyses: race/ethnicity, licensure status, education, primary discipline, and number of learned EBPs. We also included a categorical variable that identified whether an EBP had structured content as an EBP-level covariate to control for EBP content differences (Barnett et al., 2017). All variables were centered at the grand mean prior to calculating interaction terms.

A second, exploratory model examining therapist-level psychological safety was conducted after testing our initial hypothesis. This variable was group-mean centered prior to calculating interaction terms (Enders & Tofighi, 2007). We added two cross-level interactions to the previous model (i.e., therapist-level psychological safety and program-level sustainment leadership and therapist-level psychological safety and program-level EBP sustainment climate). For this regression analysis with random intercepts and random slope (for therapist-level psychological safety), we used the same “mixed” command and maximum likelihood estimator in Stata. Missing data were addressed using listwise deletion, which resulted in 1.1% or 6.2% of data missing depending on the model. All analyses were completed using Stata/IC version 16 and SPSS version 26.

## Results

Table 2 presents the results of multilevel regression analyses. Model 1 presents the proposed model, but without interaction effects. In this model, only program-level sustainment leadership was found to be associated with therapists’ EBP self-efficacy (*b* = 0.35, *p* = 0.001). In model 2, which is the proposed model including interaction effects, no significant interactions were found. However, the main effects of program-level sustainment leadership and program-level psychological safety on therapists’ EBP self-efficacy were significant such that higher sustainment leadership (*b* = 0.36, *p* < 0.001) and psychologicsal safety (*b* = 0.21, *p* = 0.049) predicted higher therapist EBP self-efficacy. Effect sizes were calculated for these main effects (Selya et al., 2012) and both variables had the same small-to-medium effect, *f^2^* = 0.077 (Lorah, 2018). However, program-level EBP sustainment climate was not associated with therapist-reported EBP self-efficacy.

**Table 2.** Regression results predicting therapist EBP self-efficacy for models with direct effects only (model 1), program-level psychological safety (model 2) and therapist-level psychological safety (model 3).

Variables	Model 1				Model 2				Model 3			
	*b*	SE	* p*	95% CI	*b*	SE	* p*	95% CI	*b*	SE	* p*	95% CI
Controls												
Number of EBPs ever used	.07	.03	.018*	[.01, 13]	.07	.03	.017*	[.01,.13]	.07	.03	.030*	[.01,.13]
Licensure Status	.18	.07	.014*	[.04,.33]	.18	.07	.014*	[.04,.33]	.18	.08	.016*	[.03,.33]
Race (Hispanic)	.34	.09	.000***	[.16,.51]	.34	.09	.000***	[.16,.52]	.35	.09	.000***	[.17,.53]
Race (Other)	.25	.10	.014	[.05, 45]	.26	.10	.012*	[.06,.46]	.27	.10	.010*	[.06,.47]
Discipline (Social work)	-.15	.08	.057	[-.29,.00]	-.14	.08	.071	[-.29,.01]	-.12	.08	.137	[-.27,.04]
Discipline (Counseling & Psychology)	-.04	.12	.757	[-.27,.20]	-.03	.12	.797	[-.27,.21]	-.01	.12	.957	[-.25,.24]
Structured EBP	.15	.05	.004**	[.05,.25]	.15	.05	.004**	[.05,.25]	.16	.05	.003**	[.05,.26]
Direct Effects												
Sustainment Leadership	.35	.10	.001**	[.15,.55]	.36	.10	.000*	[.16,.57]	.35	.11	.001**	[.15,.56]
Sustainment Climate	-.08	.10	.404	[-.28,.11]	-.09	.10	.387	[-.29,.11]	-.07	.10	.493	[-.28,.13]
Program-level Psychological Safety	.19	.10	.067	[-.01,.39]	.21	.11	.049*	[.00,.42]	.18	.11	.095	[-.03,.40]
Therapist-level Psychological Safety	-	-	-	-	-	-	-	-	.08	.06	.151	[-.03,.21]
Interactions												
Sustainment Leadership × Program-level Psychological Safety	-	-	-	-	.09	.14	.490	[-.17,.36]	.11	.14	.440	[-.17,.38]
Sustainment Climate × Program-level Psychological Safety	-	-	-	-	-.05	.23	.839	[-.50,.41]	-.11	.24	.656	[-.58,.37]
Sustainment Leadership × Therapist-level Psychological Safety	-	-	-	-	-	-	-	-	.39	.17	.021*	[.06,.72]
Sustainment Climate × Therapist-level Psychological Safety	-	-	-	-	-	-	-	-	-.36	.18	.051	[-.72,.00]

*p < .05, **p < .01, ***p < .001.

In model 3, our exploratory model including therapist-level psychological safety, the interaction between program-level sustainment leadership and therapist-level psychological safety was significant (*b* = 0.39, *p* *=* 0.021), but no other interactions with sustainment leadership or climate were significant. The interaction also had a small-to-medium effect (*f^2^* = 0.076). The main effect of program-level EBP sustainment climate remained significant as well (*b* = 0.35, *p* *=* 0.001), but program-level psychological safety was no longer significant. Similar to the previous model, the main effect of program-level EBP sustainment climate was not significant.

**Figure 2.**
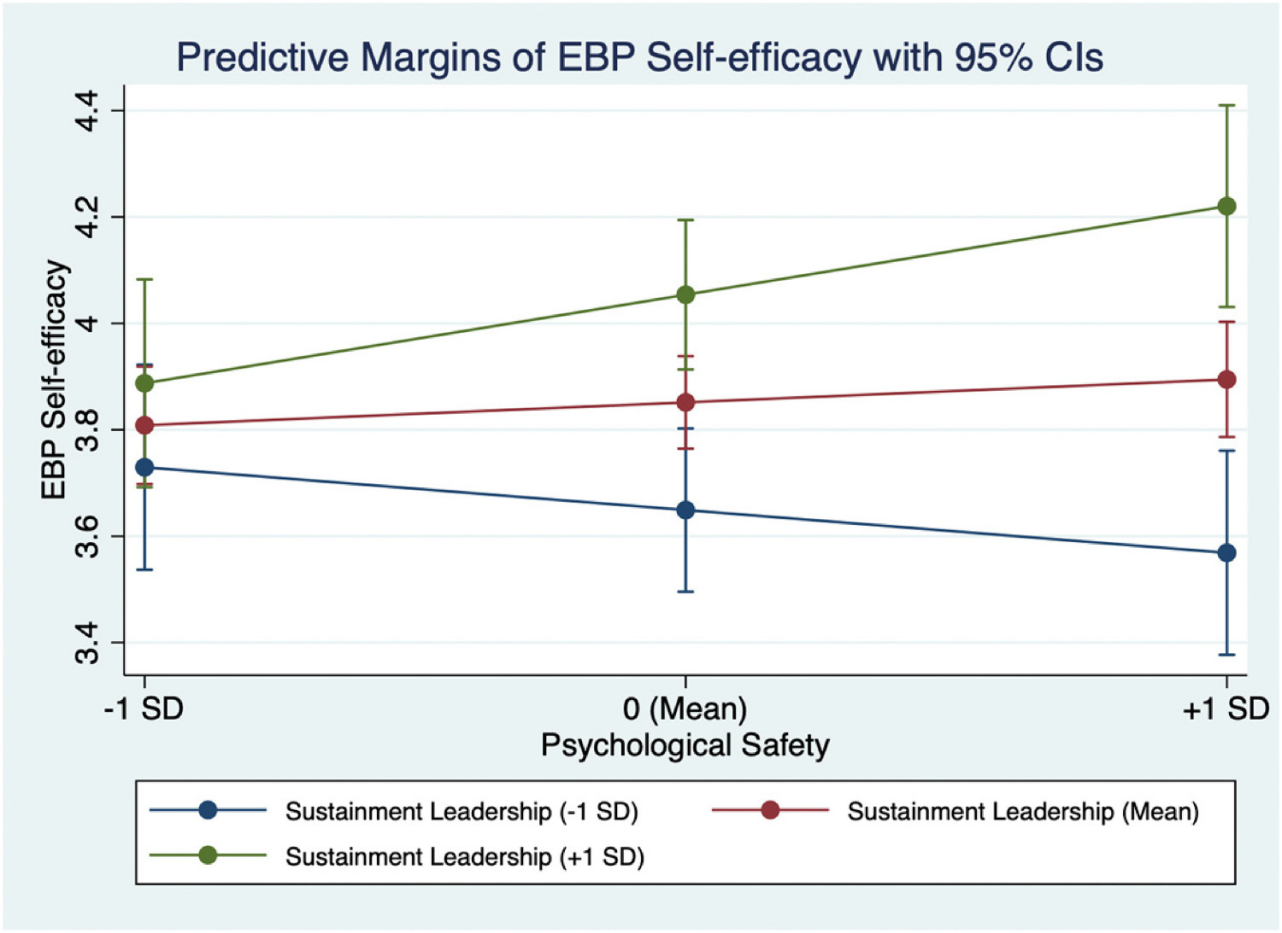
Simple slopes of therapist-level psychological safety predicting EBP self-efficacy for low (−1 SD), medium (mean), and high (+ 1 SD) levels of sustainment leadership.

To further probe the significant cross-level interaction, we conducted post hoc simple slope analyses. This analysis revealed that the conditional effect of psychological safety on EBP self-efficacy was significant at high levels (one SD above the mean) of sustainment leadership (dy/dx = 0.31., *p* *=* 0.013), but not at low (one SD below the mean) or average levels (at the mean of programs). (See Figure 2).

In terms of model covariates, in both models 2 and 3 higher number of EBPs being used by therapists (*p* = 0.030) and professional licensure were positively associated with self-efficacy (*p* *=* 0.016). Also, therapists who identified as Hispanic (*p* < 0.001) or as a member of another ethnic minority group (*p* = 0.012) reported higher EBP self-efficacy than therapists who identified as White. Additionally, therapists reported higher EBP-efficacy for structured EBPs compared to those that are unstructured (*b* = 0.16, *p* = 0.003). There were no differences in reported self-efficacy based on therapists’ primary discipline (e.g., social work, counseling, psychology, or marriage and family therapy).

## Discussion

As public mental health systems make costly investments in EBP implementation efforts, there is utility in examining conditions that relate to on-going EBP sustainment to inform targeted improvements. In this paper, we were interested in examining how organizational sustainment leadership and climate may interact with psychological safety to create conditions conducive to high levels of EBP self-efficacy, an important predictor of sustained EBP use. The current study examined how psychological safety may moderate the effects of organizational sustainment leadership and sustainment climate on therapists’ EBP self-efficacy in the context of a system-driven implementation of multiple EBPs in children's mental health.

In our initial analyses, program-level sustainment leadership and psychological safety showed associations with therapist self-efficacy, but no significant interactions emerged. Although our original hypothesis was unsupported, we conducted an additional exploratory analysis examining individual (or therapist)-level psychological safety as the factor that may more proximally relate to outcomes of interest. In community mental health clinics, therapists mostly work with clients individually rather than in teams and perceptions of psychological safety may vary considerably across staff members. In our exploratory analysis, we found a significant interaction between program-level sustainment leadership and therapist-level psychological safety. Given the nature of this multi-level moderation analysis, therapist-level psychological safety cannot be interpreted as moderating the higher-level relationship between program-level sustainment leaderhip and EBP self-efficacy (Preacher et al., 2016). Instead, we may infer only that the positive link between therapist's sense of psychological safety and EBP self-efficacy only held in programs characterized by high sustainment leadership. Thus, although we did not obtain support for our a priori hypothesis that organizational psychological safety was a precondition for positive effects of sustaintment leadership and climate, we did find evidence that perceived psychological safety promoted self-efficacy when leadership was strong in championing EBP implementation.

Overall, we noted independent links between organizational sustainment leadership and organizational psychological safety and therapists feelings of confidence and mastery self-efficacy with EBPs. Thus, sustainment leadership and psychological safety both represent potentially important and distinct targets of organizational interventions but it may not be critical to assess for workplace psychological safety before deploying organizational leadership strategies. Again, therapists’ individual perceptions of psychological safety were linked to self-efficacy only in programs with high sustainment leadership.

Our findings suggests that organizations with program leaders who are knowledgeable about, able to support the tasks associated with EBP delivery, and tenacious about EBP sustainment may be able to support therapists feelings of mastery when overall levels of psychological safety are less than optimal. We also found that sustainment leadership impacts self-efficacy above and beyond perceptions of organizational EBP sustainment climate. This may be because strong leaders are attuned to barriers to implementation and sustainment in their programs and are able to work with employees to remove these barriers. These findings point to the centrality of supporting community mental agencies to cultivate sustainment focused leadership, which may in turn increase self-efficacy in front-line therapists.

Our results differ from previous studies that describes implementation climate as potential mediator of the relationship between implementation leadership and EBP use or behavior. Although the two variables were moderately strongly correlated (Table 3), there was no evidence that the relationship between sustainment leadership and the outcome was diminished by sustainment climate. Rather, with both variables in the model, only sustainment leadership explained variance in therapist self-efficacy. A main difference in this study was our focus on therapist EBP self-efficacy as an outcome, rather than EBP use. Although self-efficacy is related to and predicts actual EBP use, these are distinct constructs that may have different relationships with organizational constructs. In the Theory of Planned Behavior, self-efficacy is theorized to be associated with intentions and behaviors, and is a precursor to both (Ajzen, 2002). Even if a therapist holds positive attitudes toward an EBP and understands that EBP use is expected, if they do not believe they are capable and prepared, adoption, implementation and sustainment may not ensue. However, given the study limitations described below, additional resarch is needed to replicate these findings prior to taking action on implementation strategy design.

**Table 3.** Correlation matrix with therapist- and program-level study variables.

Variables	1	2	3	4
1. Sustainment Leadership	-	-	-	-
2. Sustainment Climate	.68	-	-	
3. Program-level Psychological Safety	.52	.27	-	-
4. Therapist-level Psychological Safety	.03	.03	.02	-

Our findings provide some support that it may be important to attend to individual differences in perceptions of psychological safety in community mental health, in addition to organizational psychological safety. Outside of training and supervision, community therapists largely work individually to learn EBPs and provide care to their clients. The independent nature of this role is also reflected in the growing number of independent contractors, rather than salaried employees, that are hired by community mental health organizations (Beidas et al., 2016). In addition, previous work has observed that the relevant relationships that build and maintain perceptions of psychological safety may vary across workplace contexts (Edmondson & Lei, 2014). One study comparing psychological safety in medical and education settings found that psychological safety perceptions varied by small groups in hospital settings (i.e., intensive care units within medical centers) while perceptions differed by entire schools in high school settings (Edmondson et al., 2016). It is possible that psychological safety is shaped by specific salient interpersonal working relationships (e.g., direct supervisor-therapist dyads) instead of group or organization-wide dynamics in community mental health settings. Thus, individual perceptions of psychological safety may be most relevant to provider self-efficacy. When participating in group supervision and consultation, therapists’ own perceptions of interpersonal safety may determine whether they elect to engage in active EBP learning behaviors (e.g., asking questions, seeking feedback, demonstrating EBP skills at risk of making a mistake), which may impact their self-efficacy. Future research is needed to examine how individual- and organizational-level psychological safety may impact proximal learning behaviors among therapists in implementation contexts.

To develop effective strategies for improving sustainment leadership and perceived psychological safety, both leaders and providers should be engaged to assess the health and functioning of key working relationships (e.g., supervisor and therapist dyads, mid-level management and organizational leadership interactions) and groups (e.g., supervision groups, EBP training cohorts). Comprensive and systematic organizational assessments that examine the social context of organizations (such as the profiles developed for organizations in Williams et al., 2019) may be useful identifying appropriate targets and goals. Once the appropriate targets have been determined, future qualitative studies can assess the feasibility and acceptability of interventions that integrate individual- and organization-directed strategies aimed at developing supportive and knowledgeable leaders who address appropriate EBP sustainment barriers and fostering psychological safety. For example, interventions in burnout prevention in healthcare workers have been shown to be most effective when strategies are aimed at both levels (e.g., peer support groups and consultation and organizational strategies to increase therapist involvement in organizational policy decision-making) (Dreison et al., 2018; Panagioti et al., 2017). Stakeholder input in the development and implementation of these strategies will be essential to tailor them to the specific needs and context of each organization.

There are several limitations to this study that necessitate tempered conclusions and indicate important directions for future research. First, we are unable to draw conclusions about the directionality of associations because constructs were assessed in a cross-sectional analysis. We are unable to rule out alternative interpretations, for example, staff therapists’ self-efficacy with EBPs may have compelled more organizational leadership support for EBPs. It is also plausible that an unmeasured individual difference factor (e.g., neuroticism) contributes to therapists’ low confidence with EBPs, negative perceptions about their organization, and greater apprehensiveess about making mistakes and thus lower pereceived psychological safety. Future prospective studies are needed to clarify the temporal relations between psychological safety, sustainment leadership, and EBP self-efficacy. Future intervention research manipulating these organizational constructs may provide further insight into the magnitude of effects. Second, the study relied on self-report surveys to assess all constructs. Although reliance on self-report is common, this contributed to shared method variance and subjective reports of EBP-focused leadership, sustainment climate, and perceptions of psychological safety, and self-efficacy. Future studies may benefit from collection of multi-informant multi-method data, including direct observation of EBP leadership behavior and therapist implementation behavior. Furthermore, the present study used modified and shortened versions of scales that have not been validated within multi-method, multi-informant studies. Replication of findings from this study using complete, validated scales within prospective designs is needed to guide future implementation and sustainment efforts. Third, although our survey response rate of 41.1% is acceptable for surveys with community mental health therapists, (Hawley et al., 2009) it leaves room for concerns about response bias and generalizability. Finally, the present study examined provider self-efficacy with EBP delivery as an outcome, and this is not equivalent to EBP use. While self-efficacy is a theorized predictor to behavior change, future studies should prospectively examine whether and how organizational sustainment leadership, climate, and psychological safety impact sustained EBP use and fidelity among community therapists.

Despite these limitations, this study contributes new data to our emerging understanding of how therapist and organizational factors may individually and jointly impact EBP implementation in community mental health settings. In particular, organizational perceptions of sustainment leadership and psychological safety were identified as predictors of EBP self-efficacy, and there was some evidence that individual perceptions of psychological safety were associated with EBP self-efficacy only when there was sufficiently high EBP sustainment leadership support. After further study and replication, implementation scientists and organizational leaders may consider how to prioritize implementation strategies related to leadership and climate to optimize the impact of costly system-driven implementation efforts.

## References

[bibr1-26334895221110263] Aarons G. A. Ehrhart M. G. Farahnak L. R. (2014). The implementation leadership scale (ILS): Development of a brief measure of unit level implementation leadership. Implementation Science, 9(1), 45. 10.1186/1748-5908-9-45 24731295 PMC4022333

[bibr2-26334895221110263] Aarons G. A. Ehrhart M. G. Moullin J. C. Torres E. M. Green A. E. (2017). Testing the leadership and organizational change for implementation (LOCI) intervention in substance abuse treatment: A cluster randomized trial study protocol. Implementation Science, 12(1), 29. 10.1186/s13012-017-0562-3 28253900 PMC5335741

[bibr3-26334895221110263] Aarons G. A. Glisson C. Green P. D. Hoagwood K. Kelleher K. J. Landsverk J. A., & The Research Network on Youth Mental Health. (2012). The organizational social context of mental health services and clinician attitudes toward evidence-based practice: A United States national study. Implementation Science, 7(1), 56. 10.1186/1748-5908-7-56 22726759 PMC3444886

[bibr4-26334895221110263] Aarons G. A. Hurlburt M. Horwitz S. M. (2011). Advancing a conceptual model of evidence-based practice implementation in public service sectors. Administration and Policy in Mental Health and Mental Health Services Research, 38(1), 4–23. 10.1007/s10488-010-0327-7 21197565 PMC3025110

[bibr5-26334895221110263] Aarons G. A. Sommerfeld D. H. (2012). Leadership, innovation climate, and attitudes toward evidence-based practice during a statewide implementation. Journal of the American Academy of Child and Adolescent Psychiatry, 51(4), 423–431. 10.1016/j.jaac.2012.01.018 22449648 PMC3841107

[bibr6-26334895221110263] Aarons G. A. Sommerfeld D. H. Walrath-Greene C. M. (2009). Evidence-based practice implementation: the impact of public versus private sector organization type on organizational support, provider attitudes, and adoption of evidence-based practice. Implementation Science, 4(1), 83. 10.1186/1748-5908-4-83 20043824 PMC2813227

[bibr7-26334895221110263] Ajzen I. (2002). Perceived behavioral control, self-efficacy, locus of control, and the Theory of Planned Behavior. Journal of Applied Social Psychology, 32(4), 665–683. 10.1111/j.1559-1816.2002.tb00236.x

[bibr8-26334895221110263] Baer M. Frese M. (2003). Innovation is not enough: Climates for initiative and psychological safety, process innovations, and firm performance. Journal of Organizational Behavior, 24(1), 45–68. 10.1002/job.179

[bibr9-26334895221110263] Barnett M. Brookman-Frazee L. Regan J. Saifan D. Stadnick N. Lau A. (2017). How intervention and implementation characteristics relate to community therapists’ attitudes toward evidence-based practices: A mixed methods study. Administration and Policy in Mental Health and Mental Health Services Research, 44(6), 824–837. 10.1007/s10488-017-0795-0 28236076 PMC5568987

[bibr10-26334895221110263] Beidas R. S. Marcus S. Aarons G. A. Hoagwood K. E. Schoenwald S. Evans A. C. Hurford M. O. Hadley T. Barg F. K. Walsh L. M. Adams D. R. Mandell D. S. (2015). Predictors of community therapists’ use of therapy techniques in a large public mental health system. JAMA Pediatrics, 169(4), 374–382. 10.1001/jamapediatrics.2014.3736 25686473 PMC4420189

[bibr11-26334895221110263] Beidas R. S. Stewart R. Wolk C. B. Adams D. Marcus S. C. Evans A. Jackson K. Neimark G. Hurford M. Erney J. Rubin R. Hadley T. R. Barg F. Mandell D. S. (2016). Independent contractors in public mental health clinics: implications for evidence-based practices and beyond. Psychiatric Services (Washington, D.C.), 67(7), 710–717. 10.1176/appi.ps.201500234 26927579 PMC4930412

[bibr12-26334895221110263] Beidas R. S. Williams N. J. Becker-Haimes E. M. Aarons G. A. Barg F. K. Evans A. C. Jackson K. Jones D. Hadley T. Hoagwood K. Marcus S. C. Neimark G. Rubin R. M. Schoenwald S. K. Adams D. R. Walsh L. M. Zentgraf K. Mandell D. S. (2019). A repeated cross-sectional study of clinicians’ use of psychotherapy techniques during 5 years of a system-wide effort to implement evidence-based practices in Philadelphia. Implementation Science, 14(1), 67. 10.1186/s13012-019-0912-4 31226992 PMC6588873

[bibr13-26334895221110263] Bruns E. J. Kerns S. E. U. Pullmann M. D. Hensley S. W. Lutterman T. Hoagwood K. E. (2015). Research, data, and evidence-based treatment use in state behavioral health systems, 2001–2012. Psychiatric Services, 67(5), 496––503. 10.1176/appi.ps.201500014 26695495 PMC5107263

[bibr14-26334895221110263] Cannon-Bowers J. A. Salas E. Tannenbaum S. I. Mathieu J. E. (1995). Toward theoretically based principles of training effectiveness: A model and initial empirical investigation. Military Psychology, 7(3), 141–164. 10.1207/s15327876mp0703_1

[bibr15-26334895221110263] Collins C. J. Smith K. G. (2006). Knowledge exchange and combination: the role of human resource practices in the performance of high-technology Firms. Academy of Management Journal, 49(3), 544–560. 10.5465/amj.2006.21794671

[bibr16-26334895221110263] Collins-Camargo C. Royse D. (2010). A study of the relationships among effective supervision, organizational culture promoting evidence-based practice, and worker self-efficacy in public child welfare. Journal of Public Child Welfare, 4(1), 1–24. 10.1080/15548730903563053

[bibr17-26334895221110263] de Vries H. Dijkstra M. Kuhlman P. (1988). Self-efficacy: The third factor besides attitude and subjective norm as a predictor of behavioural intentions. Health Education Research, 3(3), 273–282. 10.1093/her/3.3.273

[bibr18-26334895221110263] Dorsey S. Pullmann M. D. Deblinger E. Berliner L. Kerns S. E. Thompson K. Unützer J. Weisz J. R. Garland A. F. (2013). Improving practice in community-based settings: A randomized trial of supervision – study protocol. Implementation Science, 8(1), 89. 10.1186/1748-5908-8-89 23937766 PMC3751139

[bibr19-26334895221110263] Dreison K. C. Luther L. Bonfils K. A. Sliter M. T. McGrew J. H. Salyers M. P. (2018). Job burnout in mental health providers: A meta-analysis of 35 years of intervention research. Journal of Occupational Health Psychology, 23(1), 18–30. 10.1037/ocp0000047 27643608

[bibr20-26334895221110263] Edmondson A. C. (1999). Psychological safety and learning behavior in work teams. Administrative Science Quarterly, 44(2), 350–383. 10.2307/2666999

[bibr21-26334895221110263] Edmondson A. C. (2003). Speaking up in the operating room: How team leaders promote learning in interdisciplinary action teams. Journal of Management Studies, 40(6), 1419–1452. 10.1111/1467-6486.00386

[bibr22-26334895221110263] Edmondson A. C. Higgins M. Singer S. Weiner J. (2016). Understanding psychological safety in health care and education organizations: A comparative perspective. Research in Human Development, 13(1), 65–83. 10.1080/15427609.2016.1141280

[bibr23-26334895221110263] Edmondson A. C. Lei Z. (2014). Psychological safety: the history, renaissance, and future of an interpersonal construct. Annual Review of Organizational Psychology and Organizational Behavior, 1(1), 23–43. 10.1146/annurev-orgpsych-031413-091305

[bibr24-26334895221110263] Ehrhart M. G. Aarons G. A. Farahnak L. R. (2014). Assessing the organizational context for EBP implementation: The development and validity testing of the implementation climate scale (ICS). Implementation Science, 9(1), 157. 10.1186/s13012-014-0157-1 25338781 PMC4210525

[bibr25-26334895221110263] Ehrhart M. G. Schneider B. Macey W. H. (2014). Organizational climate and culture: An introduction to theory, research, and practice (pp. xx, 364). Routledge/Taylor & Francis Group.

[bibr26-26334895221110263] Enders C. K. Tofighi D. (2007). Centering predictor variables in cross-sectional multilevel models: A new look at an old issue. Psychological Methods, 12(2), 121–138. 10.1037/1082-989X.12.2.121 17563168

[bibr27-26334895221110263] Glisson C. Schoenwald S. K. Hemmelgarn A. Green P. Dukes D. Armstrong K. S. Chapman J. E. (2010). Randomized trial of MST and ARC in a two-level EBT implementation strategy. Journal of Consulting and Clinical Psychology, 78(4), 537–550. 10.1037/a0019160 20658810 PMC3951378

[bibr28-26334895221110263] Glisson C. Williams N. J. Hemmelgarn A. Procter E. Green P. (2016). Aligning organizational priorities with ARC to improve youth mental health service outcomes. Journal of Consulting and Clinical Psychology, 84(8), 713–725. 10.1037/ccp0000107 27253202 PMC4949090

[bibr29-26334895221110263] Hawley K. M. Cook J. R. Jensen-Doss A. (2009). Do noncontingent incentives increase survey response rates among mental health providers? A randomized trial comparison. Administration and Policy in Mental Health and Mental Health Services Research, 36(5), 343–348. 10.1007/s10488-009-0225-z 19421851 PMC2715443

[bibr30-26334895221110263] Hoagwood K. E. Olin S. S. Horwitz S. McKay M. Cleek A. Gleacher A. Lewandowski E. Nadeem E. Acri M. Chor K. H. B. Kuppinger A. Burton G. Weiss D. Frank S. Finnerty M. Bradbury D. M. Woodlock K. M. Hogan M. (2014). Scaling up evidence-based practices for children and families in New York state: toward evidence-based policies on implementation for state mental health systems. Journal of Clinical Child & Adolescent Psychology, 43(2), 145–157. 10.1080/15374416.2013.869749 24460518 PMC3954943

[bibr31-26334895221110263] Holden G. (1991). The relationship of self-efficacy appraisals to subsequent health related outcomes: A meta-analysis. Social Work in Health Care, 16(1), 53–93. 10.1300/j010v16n01_05 1839087

[bibr32-26334895221110263] Hsu M. K. Wang S. W. Chiu K. K. (2009). Computer attitude, statistics anxiety and self-efficacy on statistical software adoption behavior: An empirical study of online MBA learners. Computers in Human Behavior, 25(2), 412–420. 10.1016/j.chb.2008.10.003

[bibr33-26334895221110263] Jacobs S. R. Weiner B. J. Bunger A. C. (2014). Context matters: Measuring implementation climate among individuals and groups. Implementation Science, 9(1), 46. 10.1186/1748-5908-9-46 24742308 PMC4012549

[bibr34-26334895221110263] Lau A. S. Brookman-Frazee L. (2016). The 4KEEPS study: Identifying predictors of sustainment of multiple practices fiscally mandated in children’s mental health services. Implementation Science, 11(1), 31. 10.1186/s13012-016-0388-4 26956621 PMC4784305

[bibr35-26334895221110263] Lau A. S. Lind T. Crawley M. Rodriguez A. Smith A. Brookman-Frazee L. (2020). When do therapists stop using evidence-based practices? Findings from a mixed method study on system-driven implementation of multiple EBPs for children. Administration and Policy in Mental Health and Mental Health Services Research, 47(2), 323–337. 10.1007/s10488-019-00987-2 31720914 PMC7237196

[bibr36-26334895221110263] LeBreton J. M. Senter J. L. (2008). Answers to 20 Questions about interrater reliability and interrater agreement. Organizational Research Methods, 11(4), 815–852. 10.1177/1094428106296642

[bibr37-26334895221110263] Lind T. Lau A. S. Cox J. R. Chlebowski C. Lui J. Motamedi M. Brookman-Frazee L. (2021, November 18). *Predictors of therapist delivery of evidence-based strategies in a multiple EBP implementation context*. Annual Convention of the Association of Behavioral and Cognitive Therapies, New Orleans, LA, USA.

[bibr38-26334895221110263] Lorah J. (2018). Effect size measures for multilevel models: Definition, interpretation, and TIMSS example. Large-Scale Assessments in Education, 6(1), 8. 10.1186/s40536-018-0061-2

[bibr39-26334895221110263] Los Angeles County Department of Mental Health (n.d.). *https://dmh.lacounty.gov/about/*. About DMH.

[bibr40-26334895221110263] Lucid L. Meza R. Pullmann M. D. Jungbluth N. Deblinger E. Dorsey S. (2018). Supervision in community mental health: Understanding intensity of EBT focus. Behavior Therapy, 49(4), 481–493. 10.1016/j.beth.2017.12.007 29937252 PMC6020167

[bibr41-26334895221110263] McLeod B. D. Cox J. R. Jensen-Doss A. Herschell A. Ehrenreich-May J. Wood J. J. (2018). Proposing a mechanistic model of clinician training and consultation. Clinical Psychology : A Publication of the Division of Clinical Psychology of the American Psychological Association, 25(3), e12260. 10.1111/cpsp.12260 PMC6353552 30713369

[bibr42-26334895221110263] Montcalm D. M. (1999). Applying Bandura’s theory of self-efficacy to the teaching of research. Journal of Teaching in Social Work, 19(1–2), 93–107. 10.1300/J067v19n01_08

[bibr43-26334895221110263] Nakamura B. J. Mueller C. W. Higa-McMillan C. Okamura K. H. Chang J. P. Slavin L. Shimabukuro S. (2014). Engineering youth service system infrastructure: Hawaii’s continued efforts at large-scale implementation through knowledge management strategies. Journal of Clinical Child & Adolescent Psychology, 43(2), 179–189. 10.1080/15374416.2013.812039 23819869

[bibr44-26334895221110263] Nembhard I. M. Edmondson A. C. (2006). Making it safe: The effects of leader inclusiveness and professional status on psychological safety and improvement efforts in health care teams. Journal of Organizational Behavior, 27(7), 941–966. 10.1002/job.413

[bibr45-26334895221110263] Pace B. T. Song J. Suvak M. K. Shields N. Monson C. M. Stirman S. W. (2020). Therapist self-efficacy in delivering Cognitive Processing Therapy in a randomized controlled implementation trial. Cognitive and Behavioral Practice, 28(3), 327–335. 10.1016/j.cbpra.2020.08.002

[bibr46-26334895221110263] Panagioti M. Panagopoulou E. Bower P. Lewith G. Kontopantelis E. Chew-Graham C. Dawson S. Marwijk H. v. Geraghty K. Esmail A. (2017). Controlled interventions to reduce burnout in physicians: A systematic review and meta-analysis. JAMA Internal Medicine, 177(2), 195–205. 10.1001/jamainternmed.2016.7674 27918798

[bibr47-26334895221110263] Powell B. J. Mandell D. S. Hadley T. R. Rubin R. M. Evans A. C. Hurford M. O. Beidas R. S. (2017). Are general and strategic measures of organizational context and leadership associated with knowledge and attitudes toward evidence-based practices in public behavioral health settings? A cross-sectional observational study. Implementation Science, 12(1), 64. 10.1186/s13012-017-0593-9 28499401 PMC5429548

[bibr48-26334895221110263] Preacher K. J. Zhang Z. Zyphur M. J. (2016). Multilevel structural equation models for assessing moderation within and across levels of analysis. Psychological Methods, 21(2), 189–205. 10.1037/met0000052 26651982

[bibr49-26334895221110263] Regan J. Lau A. S. Barnett M. Stadnick N. Hamilton A. Pesanti K. Bando L. Brookman-Frazee L. (2017). Agency responses to a system-driven implementation of multiple evidence-based practices in children’s mental health services. BMC Health Services Research, 17(1), 671. 10.1186/s12913-017-2613-5 28927407 PMC5606027

[bibr50-26334895221110263] Selya A. S. Rose J. S. Dierker L. C. Hedeker D. Mermelstein R. J. (2012). A practical guide to calculating Cohen’s f2, a measure of local effect size, from PROC MIXED. Frontiers in Psychology, 3, 111. 10.3389/fpsyg.2012.00111 22529829 PMC3328081

[bibr51-26334895221110263] Singer S. Lin S. Falwell A. Gaba D. Baker L. (2009). Relationship of safety climate and safety performance in hospitals. Health Services Research, 44(2p1), 399–421. 10.1111/j.1475-6773.2008.00918.x 19178583 PMC2677046

[bibr52-26334895221110263] Stewart R. E. Marcus S. C. Hadley T. R. Hepburn B. M. Mandell D. S. (2018). State adoption of incentives to promote evidence-based practices in behavioral health systems. Psychiatric Services, 69(6), 685–688. 10.1176/appi.ps.201700508 29493412 PMC6993599

[bibr53-26334895221110263] Wang W. Saldana L. Brown C. H. Chamberlain P. (2010). Factors that influenced county system leaders to implement an evidence-based program: A baseline survey within a randomized controlled trial. Implementation Science, 5(1), 72. 10.1186/1748-5908-5-72 20925947 PMC2972235

[bibr54-26334895221110263] Weiner B. J. Belden C. M. Bergmire D. M. Johnston M. (2011). The meaning and measurement of implementation climate. Implementation Science, 6(1), 78. 10.1186/1748-5908-6-78 21781328 PMC3224582

[bibr55-26334895221110263] Williams N. J. Beidas R. S. (2019). Annual research review: The state of implementation science in child psychology and psychiatry: A review and suggestions to advance the field. Journal of Child Psychology and Psychiatry, 60(4), 430–450. 10.1111/jcpp.12960 30144077 PMC6389440

[bibr56-26334895221110263] Williams N. J. Ehrhart M. G. Aarons G. A. Marcus S. C. Beidas R. S. (2018). Linking molar organizational climate and strategic implementation climate to clinicians’ use of evidence-based psychotherapy techniques: Cross-sectional and lagged analyses from a 2-year observational study. Implementation Science, 13(1), 85. 10.1186/s13012-018-0781-2 29940989 PMC6019309

[bibr57-26334895221110263] Williams N. J. Frank H. E. Frederick L. Beidas R. S. Mandell D. S. Aarons G. A. Green P. Locke J. (2019). Organizational culture and climate profiles: Relationships with fidelity to three evidence-based practices for autism in elementary schools. Implementation Science: IS, 14(1), 15. 10.1186/s13012-019-0863-9 30755220 PMC6373074

[bibr58-26334895221110263] Williams N. J. Glisson C. (2020). Changing organizational social context to support evidence-based practice implementation: A conceptual and empirical review. In Albers B. Shlonsky A. Mildon R. (Eds.), Implementation science 3.0 (pp. 145–172). Springer International Publishing. 10.1007/978-3-030-03874-8_6.

[bibr59-26334895221110263] Williams N. J. Wolk C. B. Becker-Haimes E. M. Beidas R. S. (2020). Testing a theory of strategic implementation leadership, implementation climate, and clinicians’ use of evidence-based practice: A 5-year panel analysis. Implementation Science, 15(1), 10. 10.1186/s13012-020-0970-7 32033575 PMC7006179

